# Intraventricular Vortex Interaction between Transmitral Flow and Paravalvular Leak

**DOI:** 10.1038/s41598-018-33648-9

**Published:** 2018-10-23

**Authors:** Daisuke Morisawa, Ahmad Falahatpisheh, Eleonora Avenatti, Stephen H. Little, Arash Kheradvar

**Affiliations:** 1 The Edwards Lifesciences Center for Advanced Cardiovascular Technology, University of California, Irvine, CA USA; 2 The Houston Methodist DeBakey Heart and Vascular Center, Houston, TX USA

## Abstract

Paravalvular leak (PVL) is a complication of transcatheter aortic valve replacement. Despite its marked clinical impact, no previous study has reported how PVL affects the intraventricular fluid dynamics. This study aims to delineate vortex interaction between PVL and transmitral flow and the influence of PVL orifice location on intraventricular fluid dynamics using Echocardiographic Particle Image Velocimetry. Three different conditions of no PVL, anterior PVL and posterior PVL were experimentally studied and clinically compared. Circulation, impulse, kinetic energy (KE) and change in KE (ΔKE) were calculated. As well, vortex formation analyses and streamline description were performed to study vortex interactions. The anterior PVL jet streamed into the LV and interfered with the transmitral flow. Posterior PVL jet formed a large clockwise vortex and collided with transmitral flow, which resulted in flow disturbance. Compared to no PVL condition, average circulation, impulse, KE and ΔKE increased in presence of PVL. In conclusion, we found that PVL jets lead to abnormal vortex formation that interfere with natural advancement of transmitral flow, and negatively affect the LV fluid dynamics parameters. PVL orifice location strongly affects the intraventricular vortex formation, and posterior PVL may have more negative effects compared to anterior PVL.

## Introduction

Transcatheter aortic valve replacement (TAVR) is currently a primary option in treatment of high-risk patients with severe aortic valve stenosis^1,2^. Despite the significant progress in procedural techniques and device innovation, paravalvular leak (PVL) still remains a clinical concern related to TAVR^3,4^. Incomplete sealing between the diseased native valve and deployed stented valve may lead to post-TAVR PVL. A recent clinical trial has reported that the incidence of moderate to severe post-TAVR PVL is about 5–10 percent^5^. Several studies also associate the presence of PVL, which is more than mild, with postoperative mortality^5–11^.

Like aortic regurgitation, significant PVL can lead to volume overload and left ventricular (LV) enlargement. Although it is well known that PVL adversely affects the LV function, no scientific study has reported how PVL affects intraventricular fluid dynamics and vortex formation. In this study, we investigated how advancement of transmitral flow into the LV is disturbed by the PVL jet. As well, we studied how the interaction between transmitral flow- and PVL-induced intraventricular vortex is altered according to the location of the PVL orifice. This study employed echocardiographic particle image velocimetry (Echo-PIV) to investigate the effects of clinical and *in vitro* PVL scenarios on different fluid dynamic parameters.

## Results

### Color Doppler imaging and CW Doppler echocardiography *in vitro*

**Figure 1:**
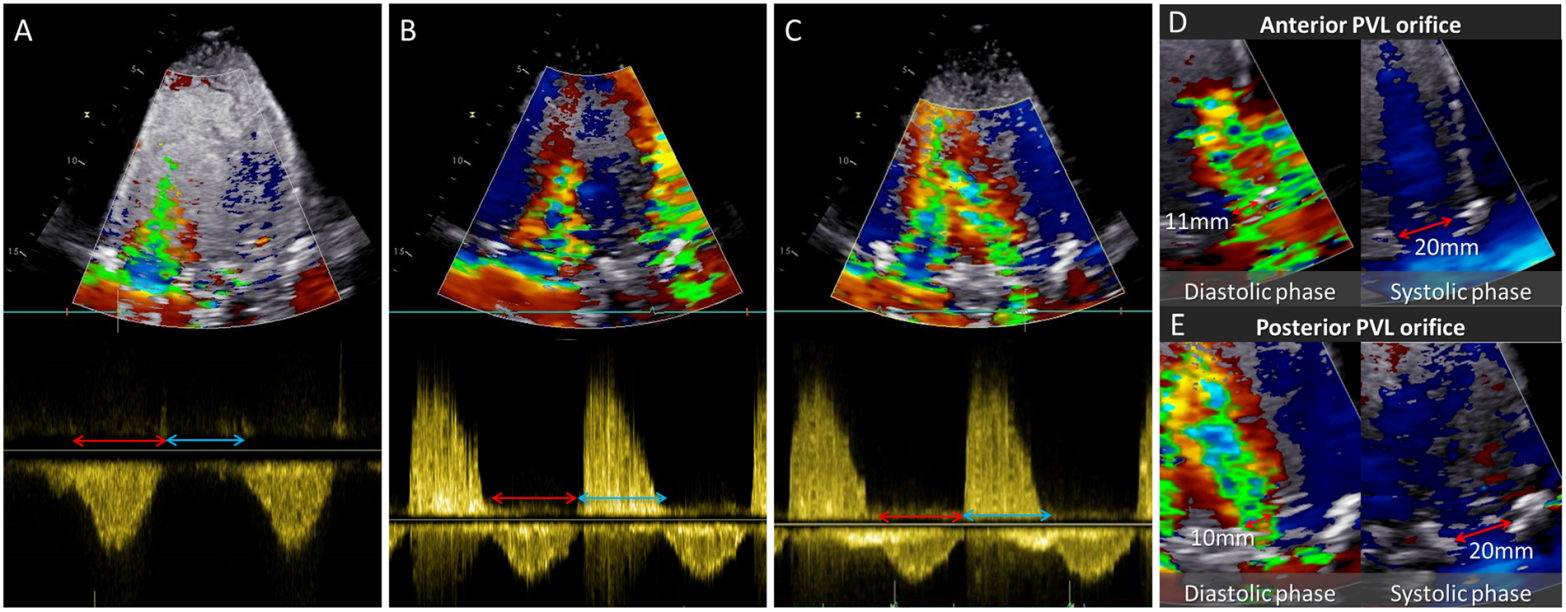
Color Doppler imaging, continuous Doppler echocardiography. (**A**–**C**) Color Doppler imaging and continuous wave (CW) Doppler echocardiography. Obvious regurgitant signal was observed in (**B**) and (**C**) and the interaction between the PVL jet and the transmitral flow was observed in (**C**). Red and blue arrows indicate systole and diastole, respectively. (**D**) and (**E**) Width of the PVL jet and outflow tract in anterior and posterior PVL case.

As expected, in the control case, no color Doppler signal denoting PVL was detected around the aortic valve during diastole (Fig. 1A). Meanwhile, apparent regurgitation signal was detected throughout diastole in experimental models of the anterior and posterior PVL (Fig. 1B,C). In presence of anterior PVL, the regurgitant jet streamed into the LV alongside the anterior wall and did not directly interfere with the transmitral flow (Fig. 1B). In posterior PVL case, the direction of PVL jet was toward the posterior wall and not along the anterior wall. Accordingly, the PVL jet had visible interaction with transmitral flow (Fig. 1C).

### Severity of paravalvular Leak

The severity of PVL was assessed based on the regurgitant volume, regurgitant fraction (RF) and ratio of PVL jet to the outflow tract width^8^. The regurgitant volumes for anterior and posterior PVL were calculated as 46 ± 0.14 ml/beat and 46 ± 0.09 ml/beat, respectively. The LV stroke volume, which corresponds to the ejected volume during systole (that also includes the regurgitant volume) was calculated as 111 ± 1.5 ml/beat and 111 ± 0.42 ml/beat, respectively. According to these values, RFs of the anterior and posterior PVL were calculated as 41.4% and 41.6%, respectively (Table 1).

**Table 1:** Severity of paravalvular leak.

Calculation of Regurgitant Volume and RF using PVL orifice area		
	Anterior PVL	Posterior PVL
PVL orifice area [cm^2^]	0.24	0.24
VTI of PVL [cm]	191 ± 0.59	192 ± 0.36
Regurgitant Volume [ml/beat]	46 ± 0.14	46 ± 0.09
Outflow tract area [cm^2^]	3.14	3.14
VTI_LVOT_ [cm]	35 ± 0.47	35 ± 0.15
SV_LVOT_ [ml/beat]	111 ± 1.5	111 ± 0.42
RF % = Regurgitant Volume/SV_LVOT_	41.4	41.6
HR [bpm]	45	45
Calculation of Regurgitant Volume and RF using the set CO		
	Anterior PVL	Posterior PVL
set CO [L/min]	3	3
HR [bpm]	45	45
SV_CIRC_ [ml/beat] = 3.0 L/45 bpm	66.7	66.7
SV_LVOT_ [ml/beat]	111 ± 1.5	111 ± 0.42
Regurgitant Volume [ml/beat] = SV_LVOT_ − SV_CIRC_	44 ± 1.5	44 ± 0.42

PVL, paravalvular leak; VTI, velocity time integral; VTI_LVOT_, VTI at left ventricular outflow tract; SV_LVOT_, stroke volume through left ventricular outflow tract; RF, regurgitant fraction; SV_CIRC_, circulating stroke volume; HR, heart rate; CO, cardiac output. The values are mean ± standard error.

We also calculated RF based on the flow condition of the heart flow simulator, the actual circulating stroke volume (ml/beat), which is the volume truly circulating in the flow loop was measured as 66.7 ml/beat (=3.0 L/min/45 bpm). The regurgitant volumes and RFs of the anterior and posterior PVL were calculated as 44 ± 1.5 ml, 44 ± 0.42 ml and 39.9% and 39.9%, respectively (Table 1).

**Figure 2:**
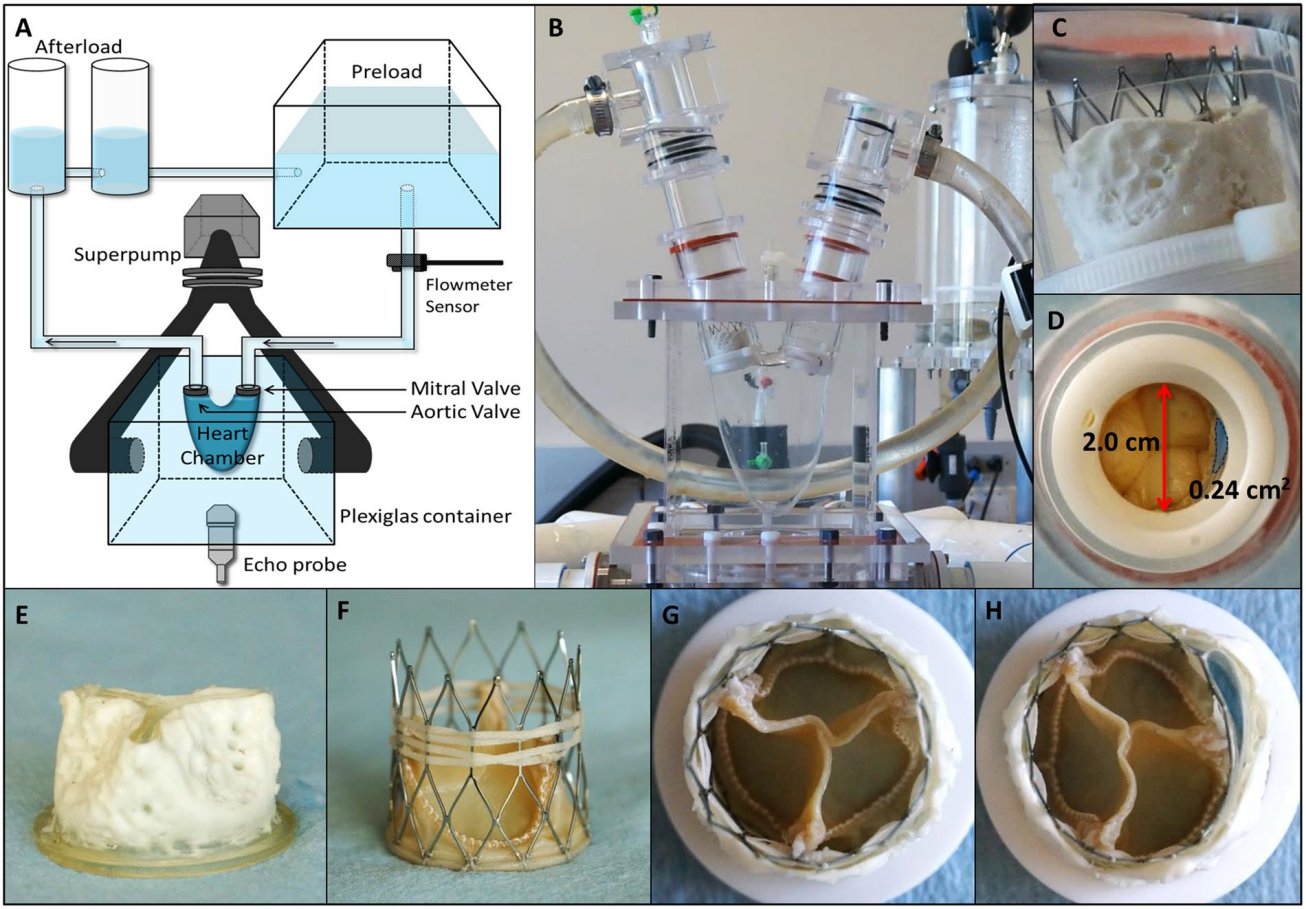
Heart flow simulator and valve models. (**A**–**C**) Experimental setup. (**D**) Paravalvular leak (PVL) orifice area and diameter of the outflow tract. (**E**) Calcified polymeric valve (**F**) FoldaValve (**G**) FoldaValve implanted in calcified polymeric valve (**H**) FoldaValve implanted in calcified polymeric valve with PVL orifice.

The severity of PVL was also assessed by calculating the ratio of PVL jet width to the width of outflow tract. In color Doppler imaging, the jet width of the anterior and posterior PVL jets were measured as 11 mm and 10 mm, respectively (Fig. 1D,E). Based on the size of the LVOT (Fig. 2D,E), the ratio of PVL jet width to the width of outflow tract was calculated as 55% and 50%, respectively. According to the ASE guideline, considering all the calculated parameters, the PVL severity was categorized as moderate in all the experiments^8,12,13^.

### Intraventricular fluid dynamics in the experimental models

**Figure 3:**
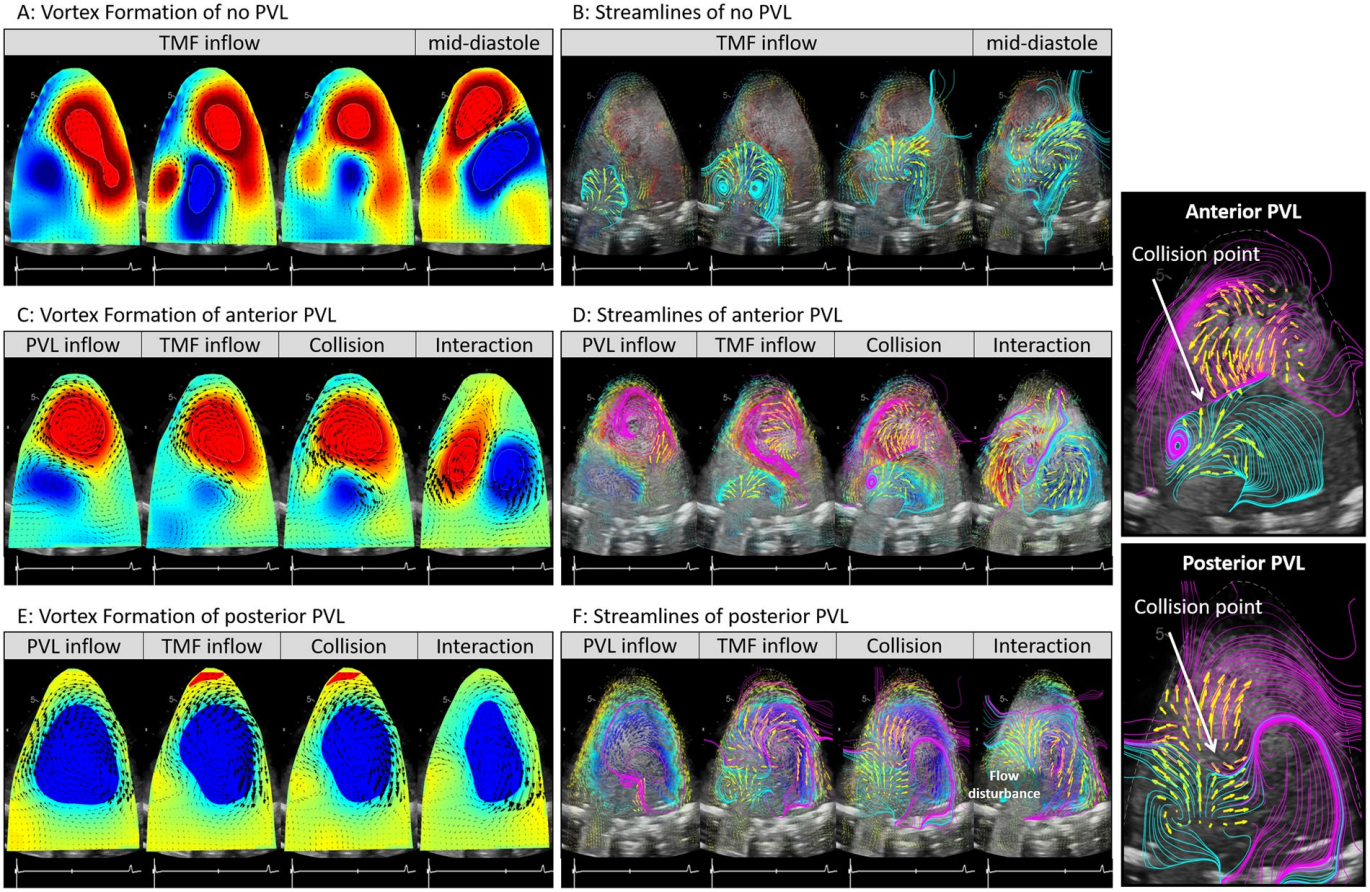
Vortices and streamlines of no PVL, anterior PVL and posterior PVL case. (**A**) Vortex formation of no PVL (**B**) streamlines of no PVL (**C**) vortex formation of anterior PVL (**D**) streamlines of anterior PVL (**E**) vortex formation of posterior PVL (**F**) streamlines of posterior PVL. TMF, transmitral flow. The collision point between the vortex formed by PVL jet and transmitral flow was observed in (**D**) and (**F**).

In the absence of PVL, the transmitral jet started flowing into LV and formed a vortex ring during early diastole (Fig. 3A,B, TMF inflow, and Video Clip 1). As the transmitral jet advanced toward the LV center, the vortex’ clockwise component moved towards the LVOT (Fig. 3A,B, mid-diastole and end-diastole, and Video Clip 1).

In the presence of anterior PVL, the PVL jet started streaming into the LV alongside the anterior wall and traveled toward the apex. The PVL jet’s boundary layers adjacent to the anterior wall formed a large counterclockwise vortex sheet at the apex during early diastole (Fig. 3C,D, PVL inflow, and Video Clip 2). Subsequently, the PVL jet’s vortex collided with the transmitral flow advancing into the LV center (Fig. 3D, TMF inflow and Collision, and Video Clip 2). After collision, the clockwise component of the transmitral vortex moved toward the LVOT, and the transmitral vortex’ counterclockwise component merged with the PVL jet’s vortex to form a large counterclockwise vortex around the mitral valve in the LV (Fig. 3C,D, Interaction, and Video Clip 2).

In posterior PVL case, the leak jet streamed into the LV, and the direction of the leak jet was toward the posterior wall (Fig. 3E,F, PVL inflow, and Video Clip 3). The leak jet made small flow disturbances around mitral valve, and formed a large clockwise vortex sheet at the LV center (Fig. 3E,F, PVL inflow, and Video Clip 3). Subsequently, the transmitral flow advanced into the LV, and collided with the large vortex sheet formed by the PVL jet (Fig. 3E,F, TMF inflow and Collision, and Video Clip 3). After the collision, large flow disturbances were observed around the mitral valve, and the large clockwise vortex was deformed (Fig. 3E,F, Interaction, and Video Clip 3). In mid- to late-diastole, the large clockwise vortex eventually absorbed the flow disturbances, and that large vortex was observed throughout cardiac cycle (Fig. 3E,F, end-diastole and end-systole, and Video Clip 3).

### Quantitative analysis of the circulation

**Figure 4:**
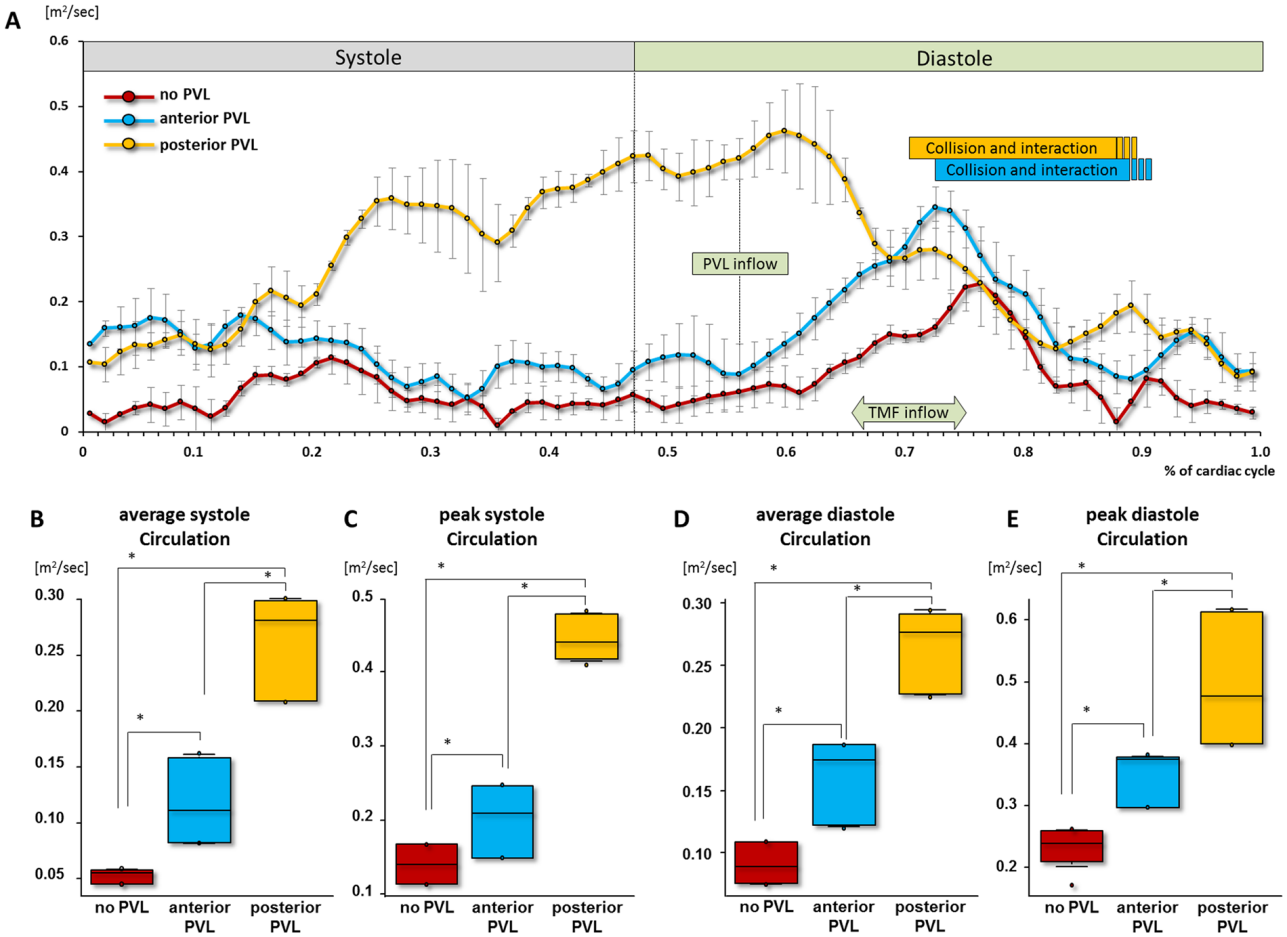
Quantitative assessment of circulation. (**A**) The time course of circulation throughout cardiac cycle. (**B**–**E**) Comparison of average and peak circulation between no PVL, anterior and posterior PVL. Red, blue and yellow depict no-PVL, anterior PVL and posterior PVL, respectively. *p < 0.05.

In the absence of PVL, circulation gradually increased during early diastole and peaked at the mid diastole in which transmitral flow was clearly visible. (Fig. 4A, red line). In presence of anterior PVL, after PVL inflow, circulation was dramatically increased until the collision of PVL and transmitral flow, and subsequently, started declining during the interaction between PVL and transmitral flow (Fig. 4A, blue line). In the presence of posterior PVL, circulation showed higher value at end-systole than those of no PVL and anterior PVL. The maximum circulation was observed just after the PVL inflow, and then circulation decreased until the end-diastole (Fig. 4A, yellow line). The average and peak circulation in both systole and diastole were in the order of posterior PVL> anterior PVL> no PVL (average circulation in systole: 0.44 ± 0.029 vs. 0.20 ± 0.043 vs. 0.14 ± 0.023, peak circulation in systole: 0.26 ± 0.042 vs. 0.12 ± 0.034 vs. 0.053 ± 0.0060, average circulation in diastole: 0.26 ± 0.030 vs. 0.16 ± 0.030 vs. 0.091 ± 0.015, peak circulation: 0.50 ± 0.010 vs. 0.35 ± 0.040 vs. 0.23 ± 0.030 m^2^/sec, p < 0.05 in all comparisons, Fig. 4B–E).

### Quantitative analysis of the impulse

**Figure 5:**
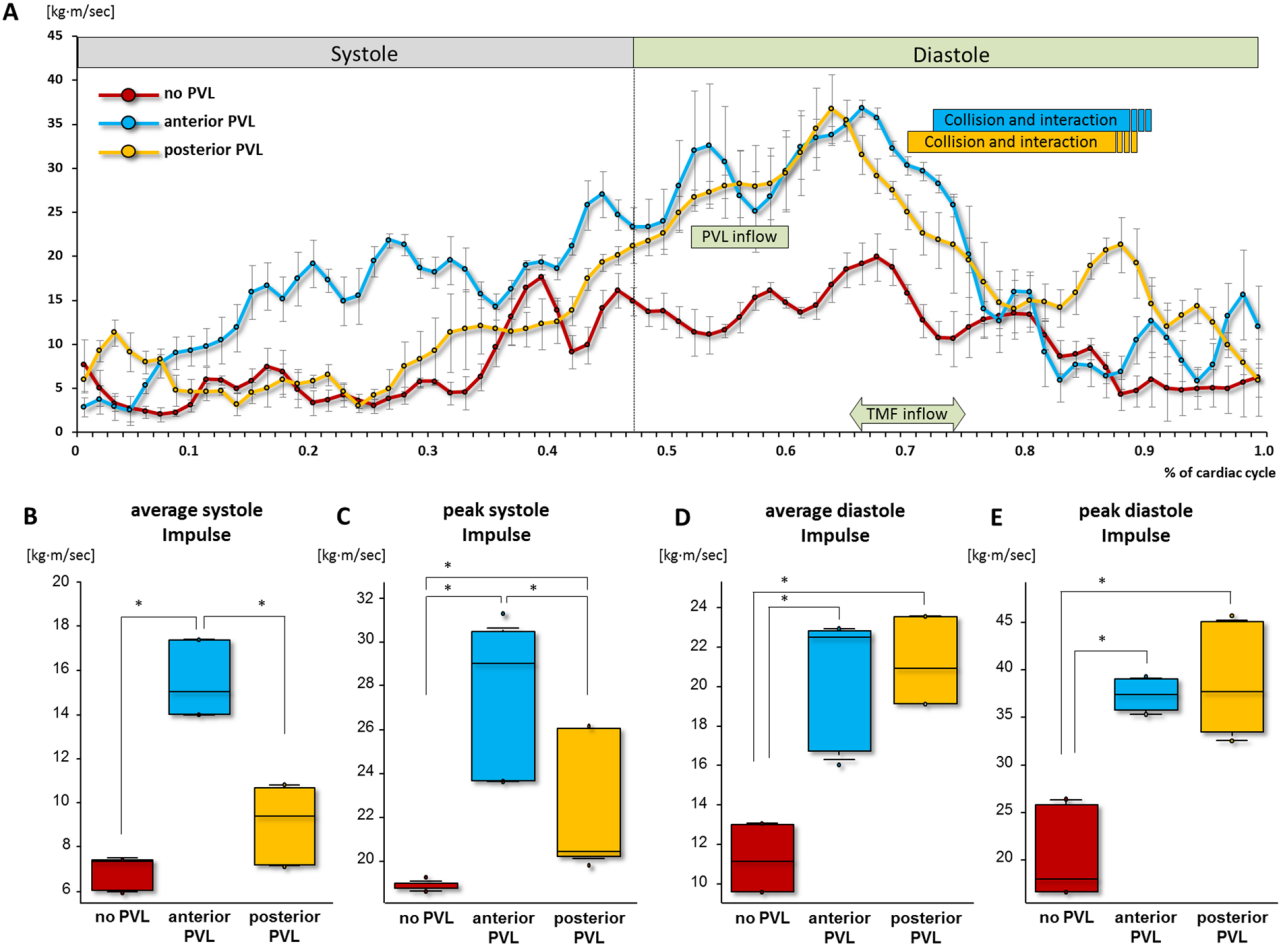
Quantitative assessment of impulse. (**A**) The time course of impulse throughout cardiac cycle. (**B**–**E**) Comparison of average and peak impulse between no PVL, anterior and posterior PVL. Red, blue and yellow depict no-PVL, anterior PVL and posterior PVL, respectively. *p < 0.05.

The impulse peaked around the initiation of transmitral inflow, and then gradually decreased during diastole in all experimental conditions (Fig. 5A). In presence of PVL, regardless its orifice location, average and peak impulse during diastole, were larger than those in absence of PVL (Fig. 5A blue and yellow line, D E). However, no significant differences between the anterior and posterior PVL were found considering the average and peak impulse during diastole (no PVL vs. anterior PVL vs. posterior PVL, average impulse in diastole: 11 ± 1.5 vs. 20 ± 3.2 vs. 21 ± 1.9, peak impulse in diastole: 20 ± 4.5 vs. 37 ± 1.6 vs. 39 ± 5.4, p < 0.05; no PVL vs. anterior PVL, no PVL vs posterior PVL in average and peak impulse, [kg·m/sec], Fig. 5D,E). During systole, the average and peak impulse in anterior PVL was the highest among the three situations. (average impulse in systole: 15 ± 1.5 vs. 9.1 ± 1.6 vs. 6.9 ± 0.67, peak impulse in systole: 28 ± 3.2 vs. 22 ± 2.9 vs. 19 ± 0.22 kg·m/sec, p < 0.05 in all comparisons, except for no PVL vs. posterior PVL in average impulse, Fig. 5B,C). Unlike diastole, a significant difference between anterior and posterior PVL was observed during systole (p < 0.05).

### Quantitative analysis of the Kinetic Energy

**Figure 6:**
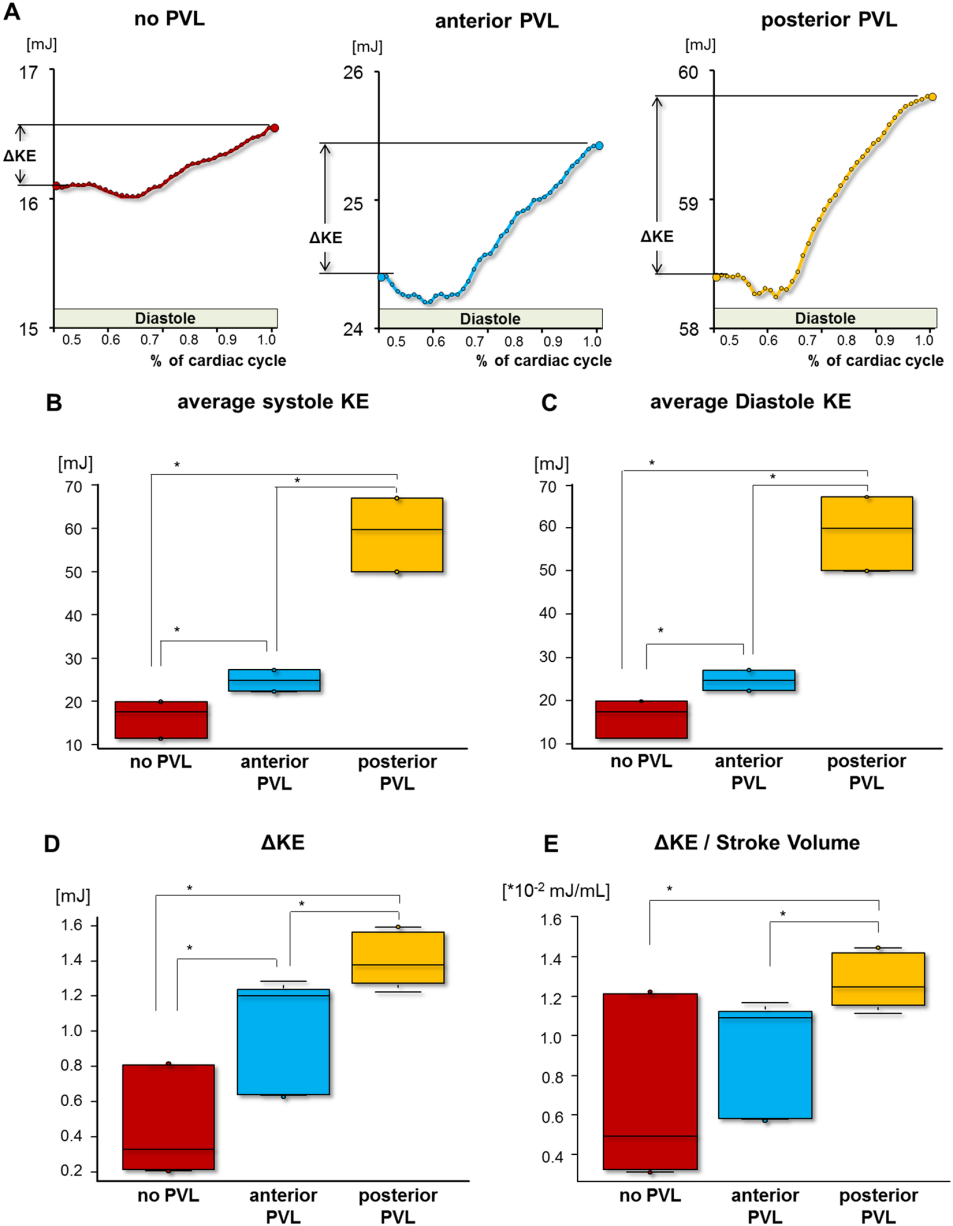
Quantitative assessment of kinetic energy. (**A**) time course of KE in the diastole part. (**B**–**E**) Comparison of average KE, ΔKE and ΔKE/stroke volume between no PVL, anterior and posterior PVL. Red, blue and yellow depict no-PVL, anterior PVL and posterior PVL, respectively. KE, kinetic energy; ΔKE, difference of kinetic energy in the end-diastole and the end-systole. *p < 0.05.

KE reduced continuously during systole, and increased through diastole in all experimental conditions. Average KE in both systole and diastole was found in order of posterior PVL> anterior PVL> no PVL (average KE in systole: 59 ± 7.4 vs. 25 ± 2.1 vs. 16 ± 3.8, average KE in diastole: 59 ± 7.4 vs. 25 ± 2.1 vs. 16 ± 3.8 mJ, p < 0.05 in all comparisons, Fig. 6B,C). As well, ΔKE was in order of posterior PVL> anterior PVL> no PVL (1.4 ± 0.15 vs. 1.0 ± 0.30 vs. 0.45 ± 0.23 mJ, p < 0.05, Fig. 6A,D). ΔKE/SV in posterior PVL case was larger than no PVL and anterior PVL (p < 0.05). As well, there were no statistically significant difference between no PVL and anterior PVL (no PVL vs. anterior PVL vs. posterior PVL: 0.67 ± 0.42 vs. 0.93 ± 0.27 vs. 1.27 ± 0.14, [×10^−2^ mJ]) (Fig. 6E).

### Vortex interaction in clinical cases

**Figure 7:**
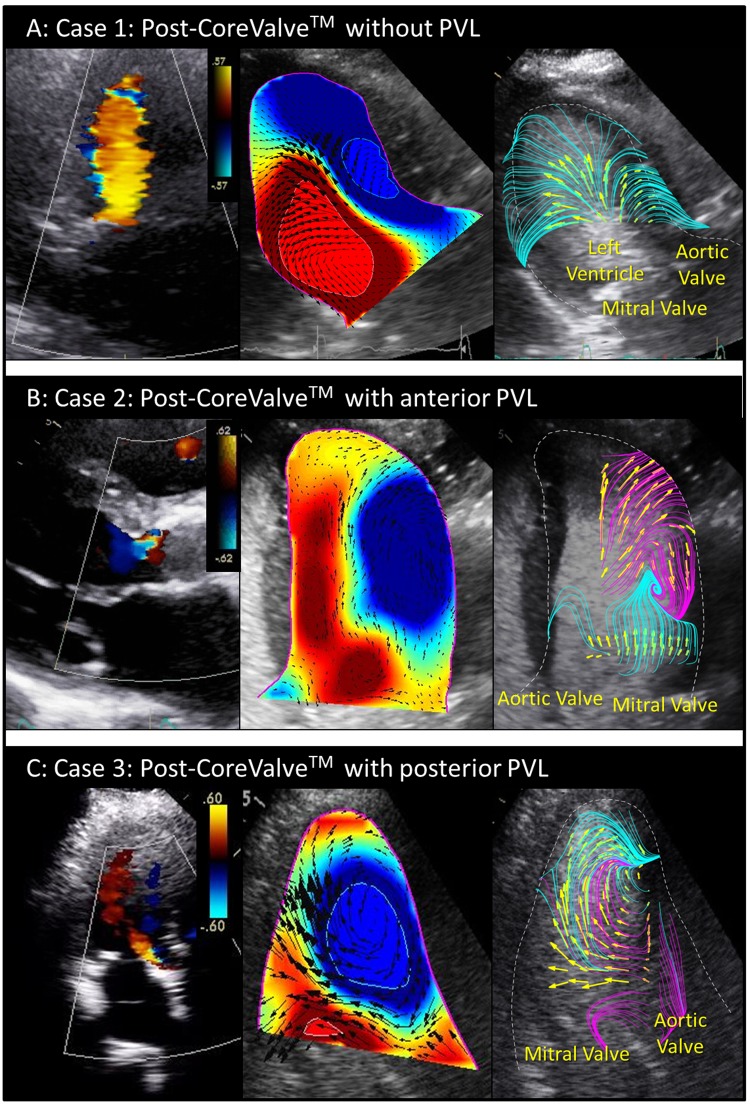
Representative clinical cases. (**A**) Post-CoreValve implantation without paravalvular leak (PVL). (**B**) Post-CoreValve implantation with anterior PVL. (**C**) Post-CoreValve implantation with posterior PVL.

In cases of post-TAVR no PVL, no leak was observed in color Doppler imaging (Fig. 7A), and transmitral vortex advanced smoothly into the LV (Fig. 7A). In case of the anterior PVL, the streamline analysis showed an image that suggests the collision of transmitral flow and a clockwise vortex, possibly due to anterior PVL jet (Fig. 7B). In this case, color-Doppler imaging clearly shows the presence of anterior PVL (Fig. 7B). However, in Echo-PIV images, we could not identify the origin of the stream interfering the transmitral flow. In case of posterior PVL, transmitral flow and PVL jet both advanced into the LV during early diastole, and merged with each other, resulting in a large clockwise vortex formation at the center of LV during late diastole (Fig. 7C).

## Discussion

More recent progress towards intracardiac flow velocimetry methods^14^ has enabled us to visualize the flow stream and to analyze the intraventricular fluid dynamics, especially vortex formation in the heart^15–18^. Several studies have reported that characteristics of the intraventricular vortices are strongly associated with cardiac performance, and in particular, transmitral vortex formation plays an important role in diastolic function^15,19–22^. In this study, we quantitatively evaluated intraventricular fluid dynamics related to PVL.

In PVL cases, the jet direction can vary depending on the location of PVL orifice^6^. Accordingly, the location of PVL orifice seems to considerably affect the intraventricular fluid dynamics. Therefore, studying the relationship between the location of PVL orifice and the intraventricular fluid dynamics could lead to better understanding of the PVL pathophysiology, and improve prediction of the disease course. However, the influence of the PVL orifice location on intraventricular vortex formation, behavior of transmitral flow and LV fluid dynamics has not been thoroughly studied yet. The present work experimentally replicated and characterized different types of post-TAVR PVL and studied the specific fluid dynamics’ situations related to each.

### Vortex interaction between transmitral flow and PVL

**Figure 8:**
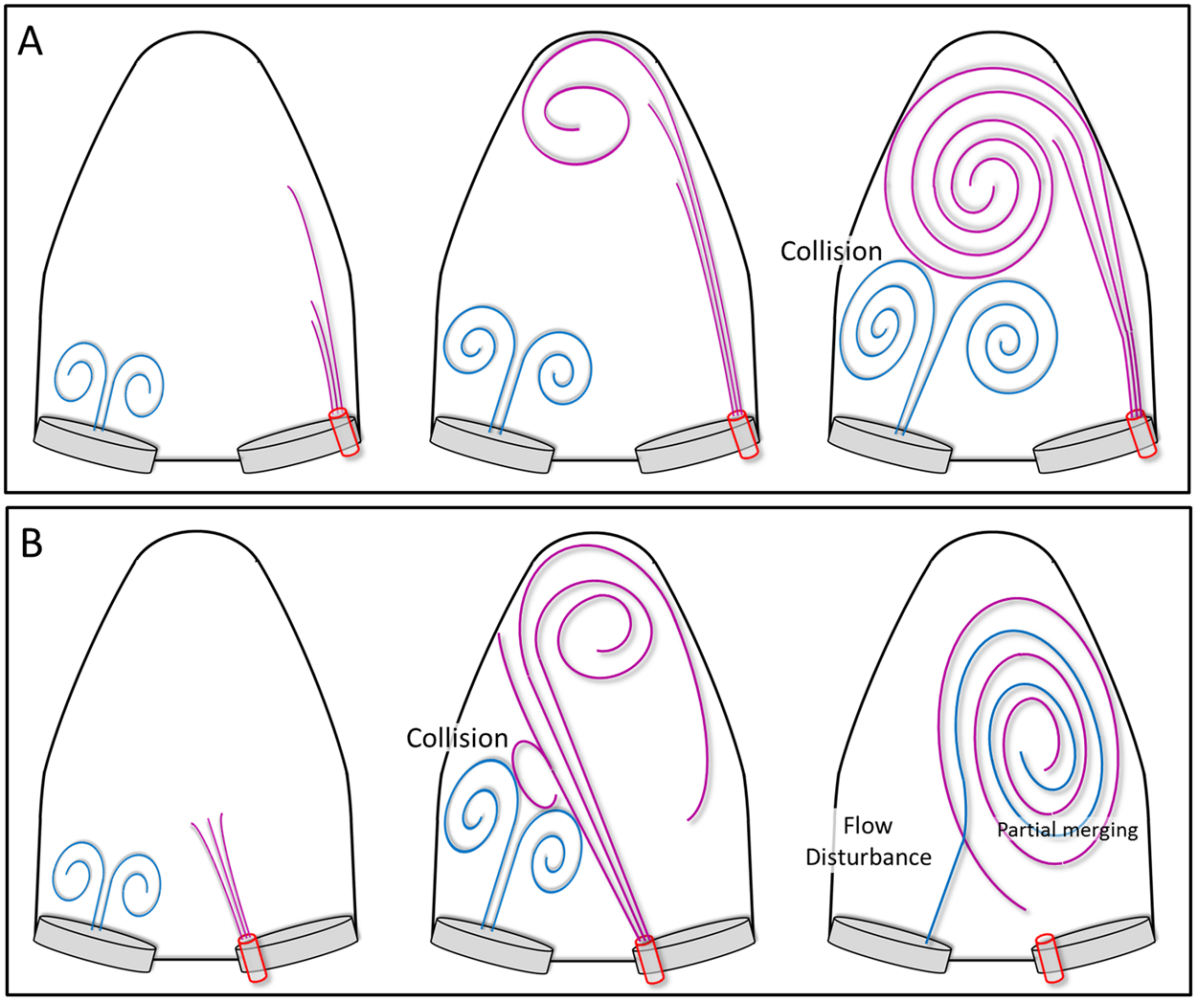
Schematics of LV vortex interaction. (**A**) Anterior PVL; the PVL jet flowed into the LV alongside the anterior wall and traveled toward the apex, and formed a large counterclockwise vortex at the apex. The PVL jet’s vortex collided with the transmitral flow. (**B**) Posterior PVL; the leak jet flow into LV traveled toward the posterior wall, and formed a large clockwise vortex sheet. The transmitral flow collided with the large vortex sheet formed by the PVL. After the collision, large flow disturbance was observed around the mitral valve in LV.

In the absence of PVL, the transmitral flow advanced to the LV without any interference, and the stream toward the LVOT was smoothly formed (Fig. 3A,B). In the anterior PVL case, although the leak jet made a large vortex at the apex, which clearly prevented transmitral flow from advancing to the center of LV, the array of vortex was like those of no PVL situation. (Fig. 3C,D). In the presence of posterior PVL, the transmitral vortex immediately collided with the large clockwise vortex formed by PVL jet, which resulted in flow disturbance around the mitral valve. (Fig. 3E,F). The vortex formation in the posterior PVL case was noticeably different from the no PVL situation. Overall, the intraventricular vortex formation was markedly different between the two experimental PVL models. These results revealed that the location of PVL orifice affects the direction of the PVL jet, and the jet direction strongly affects intraventricular vortex formation and vortex interaction between the transmitral flow and PVL jet (Fig. 8A,B).

### Quantitative analysis of Circulation

Overall, in presence of PVL, larger circulation was observed, compared to no PVL situation. In particular, posterior PVL led to the largest circulation during both systole and diastole, which seems to be due to the large clockwise vortex formed by the posterior PVL jet that lasted throughout the cardiac cycle. These results suggest that PVL inflow added the extra circulation into the LV. As well, PVL orifice location and jet direction strongly affect the intraventricular circulation.

### Quantitative analysis of Impulse

Similar to circulation, impulse was found greater in presence of PVL compared to no PVL during diastole. However, unlike circulation, no difference was found between the two types of PVL during diastole. These results suggest that the extra inflow due to the PVL contributed equally to the LV diastolic impulse, and the location of the orifice does not affect the flow momentum. Alternatively, during systole, a smaller impulse was observed due to the posterior PVL compared to anterior PVL. This result suggests that the flow disruption caused by the posterior PVL jet prohibits (or at least diminishes) the effective conversion of inflow momentum to outflow momentum.

### Quantitative analysis of Kinetic Energy

Average KE and ΔKE in the presence of PVL was greater compared to no PVL. As well, posterior PVL resulted in greater KE compared to anterior PVL. Meanwhile, ΔKE/SV was found greater only in the presence of posterior PVL compared to no PVL. In the presence of anterior PVL, no statistically-significant difference was found in comparison to no PVL. Accordingly, ΔKE/SV can be interpreted as the kinetic energy needed to eject unit volume. Considering no statistical difference between no PVL and anterior PVL in ΔKE/SV, the ΔKE difference between the two may be due to the increment in the ejected volume. Alternatively, greater ΔKE/SV in presence of posterior PVL may indicate larger KE dissipation.

### Vortex interaction in clinical cases

In case of post-TAVR without PVL, no flow interference with transmitral flow was observed, as anticipated. In the presence of anterior PVL, transmitral flow collided with a clockwise vortex, possibly due to anterior PVL jet. Although color Doppler imaging clearly shows the presence of anterior PVL, we could not identify the origin of the opposite-direction stream in Echo-PIV images, possibly due to the probe angle of the captured view plane. In the presence of posterior PVL, the inflow of the PVL jet and the large clockwise vortex were similar to those found *in vitro*. The LV fluid-dynamics of the two clinical cases of PVL were consistent with those of experimental data. Because of low temporal and spatial resolution and unstable location of view plane, less information could be inferred. Particularly, the location of the view plane was vulnerable in clinical examination due to its inherent sensitively to the echo probe that is strongly affected by the patient’s breath and heart motion.

### Effect of PVL on vortex formation time

The concept of vortex formation time (VFT) index in the LV has been originally developed to assess transmitral flow considering no other source of fluid entering the LV other than flow passing through the mitral valve. The classical definition of VFT computed from transmitral flow is^23^:

$${\rm{VFT}}=\frac{4}{\pi }(1-\beta )\frac{LVEDV}{{D}^{3}}EF$$

where *β* is the fraction of the stroke volume (SV) contributed from the atrial component of LV filling, *LVEDV* is left ventricular end diastolic volume, *D* is the effective diameter of mitral geometric orifice area and *EF* is ejection fraction. Considering that

$$LVEDV\times EF=SV,$$

VFT is calculated as:

$${\rm{VFT}}=\frac{4}{\pi }(1-\beta )\frac{SV}{{D}^{3}}$$

Classical VFT definition implies^23^:

$$SV={V}_{E}+{V}_{A}$$

where ${V}_{E}$ and ${V}_{A}$, are volumes contributed to LV during diastolic E- and A-wave, respectively. Given that in these experiments we did not consider atrial contraction phase, *SV* is equivalent to transmitral flow volume during rapid filling phase, and *β* = 0. Therefore, VFT is computed as:

$${\rm{VFT}}=\frac{4}{\pi }\frac{{V}_{E}}{{D}^{3}}$$

In this study, the actual circulating flow volume of one cardiac cycle was set to 66.7 ml, which means transmitral flow volume was the same in all the experimental condition, and since the same prosthetic valve was used for mitral position in all the experimental conditions, we may safely consider that the classical definition of VFT is not affected by the PVL. Even if the ${V}_{A}\ne 0$ and $\beta \ne 0$, the classical definition of VFT would have been equal to:

$${\rm{VFT}}=\frac{4}{\pi }(1-\beta )\frac{{V}_{E}+{V}_{A}}{{D}^{3}},$$

which is still independent of PVL volume. Overall, while PVL significantly interacts with the formation of transmitral vortex during transmitral flow, the classical definition of VFT is not affected by the PVL, as the leak jet does not affect LV suction.

### Clinical implication

In clinical practice, PVL is often underestimated because of acoustic shadowing due to calcification or prosthetic valve’s stent^24^. Therefore, careful echocardiographic examination by a skilled examiner is crucial. The present work quantitatively studied the intraventricular fluid dynamics of PVL. In that regards, we showed that the location of PVL orifice significantly influences the LV fluid dynamics. The results of our study can be potentially applied to clinical practice, and contribute to the precise assessment of PVL and its effect on the heart.

Some clinical studies have reported that larger KE and ΔKE/SV were observed in patients with low ejection fraction heart failure^25^. In the present study, greater circulation and kinetic energy were observed due to PVL. In particular, posterior PVL led to larger circulation and kinetic energy compared to anterior PVL. This means that the heart may need to consume more energy to maintain systemic circulation in presence of posterior PVL.

In the longer term, these negatively-affected fluid dynamics condition in the LV may result in deterioration of LV function, and particularly leads to systolic dysfunction. Furthermore, inappropriate vortex formation can lead to LV mechanical disadvantage since the additional momentum (due to PVL) imparted to the LV during diastole ─in the form of circulation─ results in additional kinetic energy that may increase the LV wall stress, and eventually leads to cardiomyocyte hypertrophy and fibrosis. Nevertheless, our study has only been focused on flow related phenomena and vortex interaction, and does not infer any firm conclusion about the cellular and biomechanical consequences of adverse LV flow situation.

Considering the previous clinical studies and our present results, we may safely emphasize that posterior PVL has more clinically-negative impact on intraventricular fluid dynamics. This necessitates more careful follow-up, and if needed, early therapeutic intervention that may include PVL repair. Nevertheless, further studies to delineate the effects of PVL orifice location on the conventional measures of LV diastolic function (e.g., mean diastolic gradient, E/A ratio, deceleration time, etc.) are required to establish the clinical applications.

### Study limitation

The heart flow simulator in this study only replicates diastolic rapid filling phase and not the atrial contraction. Accordingly, PW Doppler echocardiography showed only a single peak for transmitral flow indicating the absence of A-wave. Therefore, the current results only mimic the early- and mid-transmitral flow during the E-wave. The 2D echocardiographic acquisition and analysis resulted in impulse and kinetic energy to be calculated only in 2D plane and may not be valid for the whole LV. Regardless, we made sure that the echo probe was ideally positioned to fully capture LV in-flow and out-flow. Furthermore, only a moderate PVL situation was replicated in this study; however, severity of PVL may result in other fluid dynamics situations.

In the clinical cases, we studied PVL around the Medtronic’s CoreValve while in the experimental cases, we developed PVL around FoldaValve. Since we only studied the intraventricular flow, the change in prosthesis should not affect the flow phenomena insofar as the location of the PVL orifice is the same. Additionally, instead of a native bileaflet valve with papillary muscles, we used a bioprosthetic trileaflet valve at the mitral position. This change may affect intraventricular fluid dynamics, as suggested earlier^26^. Although partial similarity of fluid dynamics behavior was observed between the clinical cases and experimental results, we remain speculative that the structural differences might have affected the flow.

## Conclusions

This study found that PVL jets lead to abnormal vortex formation that clearly interfere with natural advancement of transmitral flow into the LV, and negatively affects blood’s circulation, impulse and kinetic energy. As well, the location of PVL orifice strongly affects the intraventricular vortex formation, based on the statistical comparisons between different cases. Posterior PVL may have more negative effects on the LV fluid dynamics parameters compared to anterior PVL.

## Methods

### Heart flow simulator

We used a heart flow simulator that creates pulsatile circulatory flow, as previously described^27–29^. The schematic layout and picture of the experimental setup are shown in Fig. 2A,B. The system consists of a silicone ventricular chamber immersed and pressurized in a Plexiglas container. The silicone ventricular chamber is designed based on an adult LV shape. The system is actuated by a pulsatile pump system (Superpump system, VSI, SPS3891, Vivitro systems Inc., Victoria, Canada). An ultrasonic flowmeter (Transonic Systems Inc., Ithaca, NY) was placed on the upper stream of the LV (Fig. 2A). Distilled water was used as the circulatory flow, and the pulsatile flow was generated as the response of the silicone chamber to the input waveform provided by the pump^27^. Because the accuracy of Echo-PIV may diminish in higher flow velocities, heart rate and cardiac output were set lower in the experiments^15^. Therefore, the flow conditions were set to a cardiac output of 3.0 L/min at a heart rate of 45 beats per minute (bpm) under physiological waveforms.

### Heart Valves and models of PVL

A 25 mm bioprosthetic mitral valve (Biocor, St. Jude Medical Inc., St Paul, MN) was used at the mitral position. We used our recently developed polymeric calcified valve^30^ in place of stenotic aortic valve (Fig. 2E), and FoldaValve as a transcatheter aortic valve (TAV). FoldaValve is a self-expandable aortic valve with a 25 mm nitinol stent frame made of bovine pericardial leaflets (Fig. 2F)^31^. The post-TAVR control situation was created by deploying the TAV within the polymeric calcified valve, and no PVL was ensured (Fig. 2C,G). To create post-TAVR PVL conditions, a PVL orifice was created by placing a folded plastic sheet within the gap between the TAV and the polymeric calcified valve (Fig. 2D,H). Using image processing, the PVL orifice area was measured by considering the pixel count ratio of the orifice and valve annulus.

### Experimental conditions

**Figure 9:**
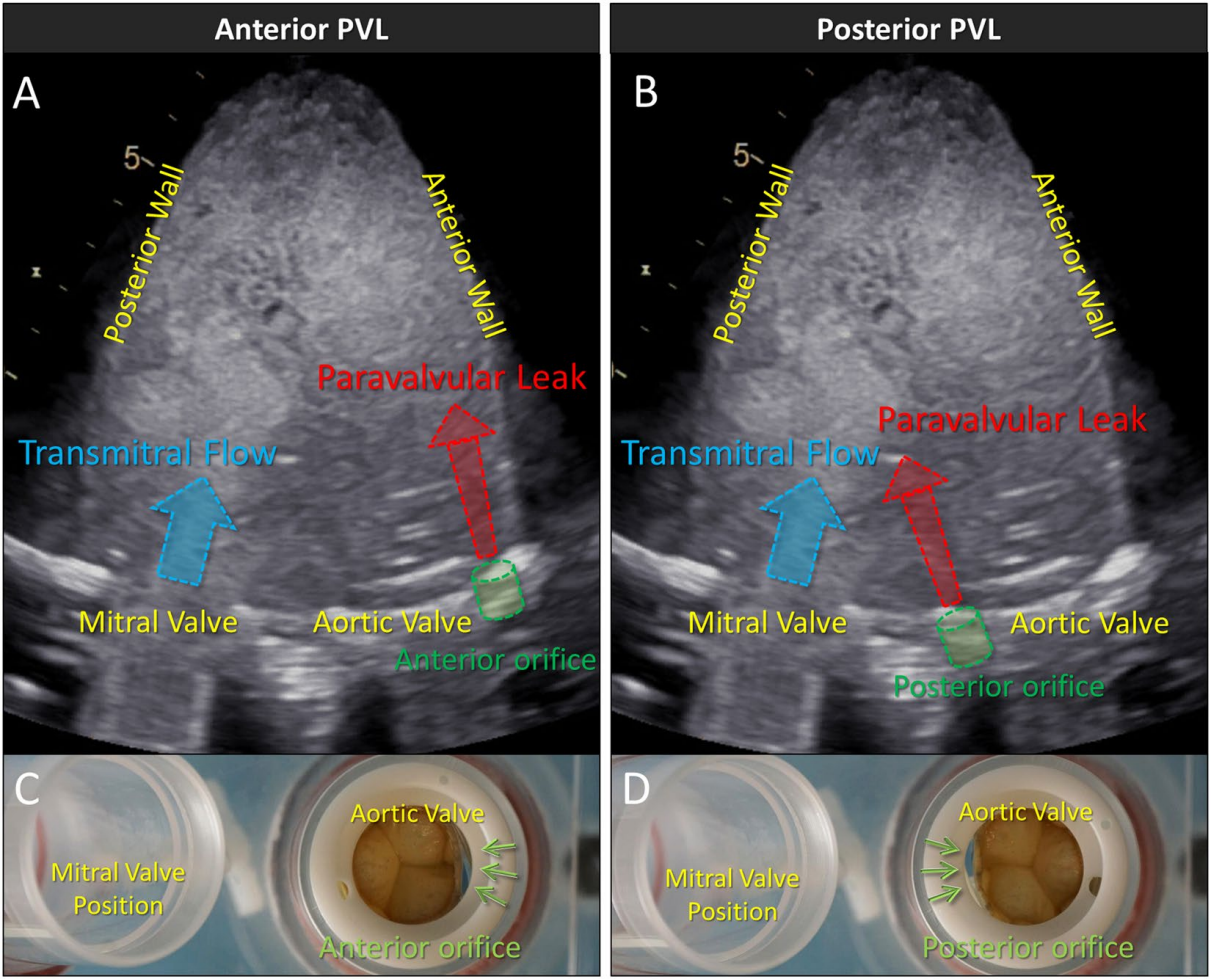
Schematics of paravalvular leak (PVL) in experiments. (**A**) and (**B**) Echocardiographic images of the LV model with anterior and posterior PVL. (**C**) and (**D**) PVL orifice in heart flow simulator.

Anterior and posterior PVL conditions were individually created by altering the valve orientation accordingly (Fig. 9C,D). Figure lA and B show the echocardiographic images indicating the location of PVL orifice at the anterior and posterior side of the aortic annulus. In total, three sets of experiments, i.e., no PVL, anterior PVL and posterior PVL, were performed to assess PVL fluid dynamics, and in particular, the interaction between the vortices formed by transmitral and PVL jets.

### Echocardiographic studies

A GE Vivid E9 echocardiography system (GE Healthcare, Milwaukee, WI) was used, and a 4VD ultrasound transducer was securely placed at the bottom of the Plexiglas container (Fig. 2A). 1 ml of the contrast agent, Optison (GE Healthcare Inc., Princeton, NJ) was injected from the upper stream of the LV, and then, 3 cardiac cycles were stored while the whole LV was appropriately enhanced by the contrast agent (Fig. 9).

### Data analysis

The obtained data was exported to a workstation, and flow analyses were performed by the dedicated software, Hyperflow (AMID, S.R.L., Sulmona, Italy). Streamlines of intraventricular flow were numerically computed and visualized by MATLAB (MathWorks, Inc., Natick, MA). Streamlines related to transmitral flow and PVL were differentiated by cyan and magenta. Clockwise and counterclockwise vortical flows are shown in blue and red, respectively.

Fluid dynamics’ parameters: circulation, impulse and kinetic energy (KE) were computed by MATLAB. Three echocardiography cine data in each experimental condition were used for the analysis of the fluid dynamics’ parameters. Each dataset consisted of three consecutive cardiac cycles. Circulation is the flux of vorticity computed as the surface integral of the curl of the velocity field normal to the area confined by a closed boundary, and is commonly used for describing the vortex’ strength^29^. Therefore, larger circulation means stronger rotational tendency in the flow. The impulse is another fluid dynamics’ parameter attributed to the flow by a jet, which indicates the flow momentum that correlates with flow mass and velocity. The impulse was calculated as follows:

$$I=\frac{1}{2}\rho {\int }_{LV}{\boldsymbol{x}}\times {\boldsymbol{\omega }}dV,$$

where *ρ* is the distilled water density used as the circulating fluid, **x** is the displacement vector, ***ω*** is the vorticity and *dV* is a differential element related to each LV voxel. Furthermore, flow kinetic energy is the direct consequence of the LV work and represents the amount of energy the fluid is subjected to when it moves. Flow kinetic energy correlates with flow velocity squared and fluid density. KE was calculated as follows:

$$KE=\frac{1}{2}\rho {\int }_{LV}{v}^{2}dV.$$

To overcome the planar nature of the acquisition, we considered a unit value depth for each pixel that the 2D Echo-PIV measured the velocity in it, treating it as a voxel. In addition, the change in KE (ΔKE; difference between end-diastolic and end-systolic KE) was computed. In presence of PVL, the stroke volume (SV) was defined as the ejected volume that includes regurgitant flow volume. Therefore, the SV in presence or absence of PVL could be different since the actual circulating flow was set as 3.0 L/min for each experimental condition. To normalize kinetic energy for each flow condition, we calculated ΔKE/SV as well.

The average value for each parameter was plotted along with the standard error bar (Figs 4A, 5A). Using these parameters, we quantitatively characterized intraventricular fluid dynamics parameters during both systole and diastole. Statistical analysis, Kruskal-Wallis and post-hoc Steel-Dwass tests were performed with EZR (Saitama Medical Center, Jichi Medical University), a modified version of R commander (version 2.3-0)^32^.

### Representative clinical cases

Three representative clinical cases (i.e., post-CoreValve implantation without PVL, and two post-CoreValve implantation with PVL) were studied. Those images were provided by the Houston Methodist DeBakey Heart and Vascular Center. All methods regarding patients’ data acquisition were carried out in accordance with the hospital guidelines and regulations. As well, all protocols were approved by the Houston Methodist Institutional Review Board and the informed consent was obtained from all subjects.

Case 1 was an 84-year-old male with a 31 mm CoreValve with no PVL observed in post-operative echocardiography. Case 2 was a 76-year-old male with a 29 mm CoreValve implant in whom a mild PVL jet from the anterior side of aortic valve was detected. Case 3 was a 73-year-old male with a 31 mm CoreValve implant and trace PVL jet from posterior side of aortic valve. All patients were originally diagnosed with severe aortic stenosis based on the ASE guideline^33^. Echocardiographic images enhanced by the contrast agent were exported to the workstation, and 2D-Echo-PIV was analyzed with Hyperflow. The streamlines were computed with MATLAB.

## Electronic supplementary material

Supplementary Information

Video Clip 1: Vortex formation with no PVL

Video Clip 2: Vortex formation in presence of anterior PVL

Video Clip 3: Vortex formation in presence of posterior PVL

## Data Availability

The datasets generated analyzed during the current study are available from the corresponding author on reasonable request.
