# Supplementary material for: Intraventricular Vortex Interaction between Transmitral Flow and Paravalvular Leak
Source: Sci Rep. 2018 Oct 23;8:15657. doi: 10.1038/s41598-018-33648-9 (PMC6199255; doi:10.1038/s41598-018-33648-9)
Supplement: Supplementary file 1 — Supplementary Information [file 41598_2018_33648_MOESM1_ESM.pdf]

Intraventricular Vortex Interaction between Transmitral Flow and Paravalvular Leak

By:

Daisuke Morisawa, M.D., Ph.D.[a] dmorisaw@uci.edu

Ahmad Falahatpisheh, Ph.D.[a] afalahat@uci.edu

Eleonora Avenatti, M.D.[b] eavenatti@houstonmethodist.org

Stephen H. Little, M.D.[b] SHLittle@houstonmethodist.org

Arash Kheradvar, M.D., Ph.D.[a] arashkh@uci.edu

a The Edwards Lifesciences Center for Advanced Cardiovascular Technology, University of California, Irvine, CA.

b The Houston Methodist DeBakey Heart and Vascular Center, Houston, TX.

Article type: Original Research Article

Address for Correspondence:

Arash Kheradvar, M.D., Ph.D., F.A.H.A.
Professor of Biomedical Engineering and Medicine
The Edwards Lifesciences Center for Advanced Cardiovascular Technology
University of California, Irvine
2410 Engineering Hall
Irvine, CA 92697-2730
Email: arashkh@uci.edu
Phone: 949-824-6538
Fax: 949-824-9968

Financial Support: National Institute of Biomedical Imaging and Bioengineering (1R21EB021513) and the American Heart Association (16IRG27250078).

VIDEO CLIPS

Video Clip 1: Vortex formation with no PVL.

The transmitral flow advanced to the LV without any interference, and the stream toward the LVOT was smoothly formed. Aortic and mitral valves are located in the left and right side, respectively, and anterior and posterior wall were located in the left and right side, respectively. Clockwise and counterclockwise vortical flow features are shown in blue and red, respectively. Flow field is shown by yellow vectors obtained from Echo-PIV.

Video Clip 2: Vortex formation in presence of anterior PVL.

The anterior PVL jet streamed into the LV alongside the anterior wall and traveled toward the apex. The PVL jet's boundary layers adjacent to the anterior wall formed a large counterclockwise vortex sheet at the apex during early diastole. Subsequently, the PVL jet's vortex collided with the transmitral flow advancing into the LV center. After collision, the clockwise component of the transmitral vortex moved toward the LVOT, and the transmitral vortex' counterclockwise component merged with the PVL jet's vortex to form a large counterclockwise vortex around the mitral valve in the LV. Aortic and mitral valves are located in the left and right side, respectively, and anterior and posterior wall were located in the left and right side, respectively. Clockwise and counterclockwise vortical flow features are shown in blue and red, respectively. Flow field is shown by yellow vectors obtained from Echo-PIV.

Video Clip 3: Vortex formation in presence of posterior PVL.

The PVL jet streamed into the LV, and the direction of the leak jet was toward the posterior wall. The PVL jet formed a large clockwise vortex sheet at the LV center. Subsequently, the transmitral flow advanced into the LV, and collided with the large vortex sheet formed by the PVL jet. After the collision, large flow disturbances were observed around the mitral valve, and the large clockwise vortex was deformed. In mid- to late-diastole, the large clockwise vortex eventually absorbed the flow disturbances, and that large vortex was observed throughout cardiac cycle. Aortic and mitral valves are located in the left and right side, respectively, and anterior and posterior wall were located in the left and right side, respectively. Clockwise and counterclockwise vortical flow features are shown in blue and red, respectively. Flow field is shown by yellow vectors obtained from Echo-PIV.
